# Ex Vivo Simultaneous H_2_^15^O Positron Emission Tomography and Magnetic Resonance Imaging of Porcine Kidneys—A Feasibility Study

**DOI:** 10.3390/jimaging10090209

**Published:** 2024-08-25

**Authors:** Maibritt Meldgaard Arildsen, Christian Østergaard Mariager, Christoffer Vase Overgaard, Thomas Vorre, Martin Bøjesen, Niels Moeslund, Aage Kristian Olsen Alstrup, Lars Poulsen Tolbod, Mikkel Holm Vendelbo, Steffen Ringgaard, Michael Pedersen, Niels Henrik Buus

**Affiliations:** 1 Comparative Medicine Lab, Department of Clinical Medicine, Aarhus University, Palle Juul-Jensens Boulevard 11, 8200 Aarhus, Denmarkchristoffer.overgaard@clin.au.dk (C.V.O.); thomasvorre@clin.au.dk (T.V.); martinbojesen@hotmail.com (M.B.); nielsmoeslund@clin.au.dk (N.M.); 2 Department of Nuclear Medicine and PET Centre, Aarhus University Hospital, Palle Juul-Jensens Boulevard 99, 8200 Aarhus, Denmark; cchmari@rm.dk (C.Ø.M.); aage.olsen@clin.au.dk (A.K.O.A.); lars.tolbod@aarhus.rm.dk (L.P.T.); mhve@biomed.au.dk (M.H.V.); 3 Department of Clinical Medicine, Aarhus University, Palle Juul-Jensens Boulevard 99, 8200 Aarhus, Denmark; 4 Department of Biomedicine, Aarhus University, Høegh-Guldbergs Gade 10, 8000 Aarhus, Denmark; 5 MR Centre, Department of Clinical Medicine, Aarhus University, Palle Juul-Jensens Boulevard 99, 8200 Aarhus, Denmark; steffen@clin.au.dk; 6 Radiology Research Unit, South Danish University, Kløvervænget 10, 5000 Odense, Denmark; 7 Department of Renal Medicine, Aarhus University Hospital, Palle Juul-Jensens Boulevard 99, 8200 Aarhus, Denmark; nhb@biomed.au.dk

**Keywords:** ex vivo kidney, normothermic machine perfusion, positron emission tomography, magnetic resonance imaging

## Abstract

The aim was to establish combined H_2_^15^O PET/MRI during ex vivo normothermic machine perfusion (NMP) of isolated porcine kidneys. We examined whether changes in renal arterial blood flow (RABF) are accompanied by changes of a similar magnitude in renal blood perfusion (RBP) as well as the relation between RBP and renal parenchymal oxygenation (RPO). Methods: Pig kidneys (n = 7) were connected to a NMP circuit. PET/MRI was performed at two different pump flow levels: a blood-oxygenation-level-dependent (BOLD) MRI sequence performed simultaneously with a H_2_^15^O PET sequence for determination of RBP. Results: RBP was measured using H_2_^15^O PET in all kidneys (flow 1: 0.42–0.76 mL/min/g, flow 2: 0.7–1.6 mL/min/g). We found a linear correlation between changes in delivered blood flow from the perfusion pump and changes in the measured RBP using PET imaging (r^2^ = 0.87). Conclusion: Our study demonstrated the feasibility of combined H_2_^15^O PET/MRI during NMP of isolated porcine kidneys with tissue oxygenation being stable over time. The introduction of H215O PET/MRI in nephrological research could be highly relevant for future pre-transplant kidney evaluation and as a tool for studying renal physiology in healthy and diseased kidneys.

## 1. Introduction

Chronic or acute impact on kidney function are very common conditions. Imaging techniques such as ultrasound and computed tomography mainly provide anatomical information [1,2]. Quantitative data on renal blood supply in terms of renal artery blood flow (RABF) or renal blood perfusion (RBP) of the parenchyma remain complicated to measure. However, these parameters are of increasing interest as they are determined by intrarenal vascular resistance and influence kidney oxygenation.

Non-invasive measurement of RABF can be performed using magnetic resonance imaging (MRI) [3], whereas estimation of RBP can be obtained with positron emission tomography (PET) and dynamic activity curves using a positron emitting tracer such as H_2_^15^O [4,5]. The blood-oxygen-level-dependent (BOLD) MRI sequence mirrors tissue deoxyhaemoglobin content, and therefore indirectly the oxygenation level [6]. RBF and oxygenation measurements have been examined in renal artery stenosis, renal allograft assessment, and renal carcinoma [7,8,9].

While H_2_^15^O PET and MRI both allow measurements of renal physiological parameters [4,10,11], a combined system has not yet been introduced in nephrological research to our knowledge. The aim of this preliminary study was to establish combined H_2_^15^O PET/MRI during ex vivo normothermic machine perfusion (NMP) of isolated porcine kidneys. We examined whether changes in RABF are accompanied by changes of a similar magnitude in RBP as well as the correlation between RBP and BOLD MRI measured renal parenchymal oxygenation (RPO).

## 2. Materials and Methods

This study was performed on four young female Danish Landrace–Yorkshire crossbred pigs with body weights of approximately 40 kg (n= 1) and 70 kg (n = 3). The study was approved by the national Danish animal research inspectorate, and all applicable institutional and national guidelines for the care and use of animals were followed. The study follows the ARRIVE reporting guidelines [12].

Animal handling and anaesthesia within our facilities have been previously described [11]. Following anaesthesia and analgesia, a 7 French sheath was placed in the femoral artery for collection of donor blood. A midline laparotomy and dissection of both kidneys was performed to expose the renal artery and vein. When both kidneys were dissected, 40.000 IE of heparin were administered iv, and 1.5–2.0 L of arterial blood were collected. Following blood collection, first the right kidney was explanted by transection of the renal artery, vein, and ureter and placed in 4 °C Ringer’s acetate, then the left kidney was explanted in a similar fashion. The donor animal was euthanized by iv injection of 20 mL pentobarbital (400 mg/mL).

For static cold storage (SCS) preparation, the kidneys were flushed with 0.5 l University of Wisconsin (UW) solution (320 mOsm/kg; [Na] 29 mEq/L; [K] 125 mEq/L). Verapamil (10 µmol/L) was added in the collected blood, the UW solution and in the saline used for flushing. Two of the seven renal arteries were extended with a 2 cm piece of ureter, which was anastomosed end-to-end with a running 7-0 suture. The renal artery was cannulated with a connector for the NMP circuit. The first kidney was connected to the NMP circuit immediately post flush. The second kidney was stored by SCS during measurements in the first kidney, approximately 2–3 h.

A total of seven kidneys were successfully removed and connected to the NMP circuit, whereas one kidney was excluded due to vascular damage during surgery making it impossible to cannulate the renal artery.

### 2.1. Ex Vivo Kidney Perfusion

**Figure 1:**
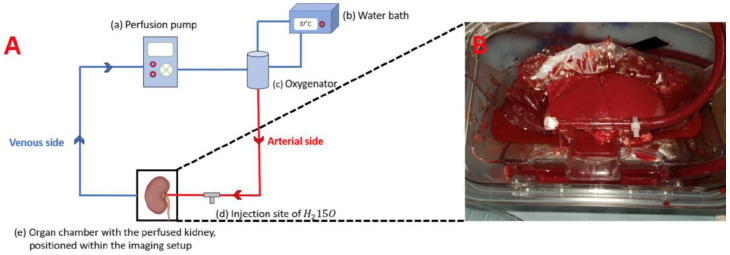
The ex vivo normothermic machine perfusion and a perfused kidney. (**A**) Schematic overview of the ex vivo normothermic machine perfusion (NMP) circuit. Blue tubes illustrate the venous side of the system, and red tubes illustrate the arterial side of the system. (**B**) Photograph illustrating the positioning of the kidney in the organ chamber. A plastic bag placed under the kidney separates the kidney from the blood in the chamber. Towels placed on the kidney are preventing it from drying up.

The kidney was perfused with a constant flow using an open NMP circuit (Figure 1).

The NMP system consisted of a pump (Medtronic Bio-Medicus 550 Bioconsole, Medtronic, Minneapolis, MN, USA), an oxygenator (Medos Hilite 1000 neonatal oxygenator, Medos, Langenselbold, Germany), and an organ chamber with the kidney being connected through the cannulated renal artery. The blood was gassed with carbogen (95% O_2_, 5% CO_2_). The system was filled with 1.5–2.0 L heparinized full blood.

Blood temperature was regulated to 37 °C by connecting a water heater (Maquet, Rastatt, Germany) to the oxygenator with an integrated heat exchanger. An infrared thermography camera (InfReC Model R500P-NNU, Avio, Yokohama, Japan) was used to monitor the temperature. All elements were connected through plastic tubes with the length of 7 m so the organ chamber could be placed in the scanning room separated from the rest of the elements of the NMP circuit in the control room. To reduce heat loss, the tubes were isolated with polyethylene foam isolation. 

The NMP system was controlled by custom build hardware (University of Groningen, The Netherlands), interfacing with a flowmeter (Transonic flowmeter, Transonic, Solana Beach, CA, USA), a pressure transducer (Edwards TrueWave, Edwards Lifescienses, Irvine, CA, USA), and the perfusion pump. By connecting the hardware to a computer with custom build software (Sophisticate, The Netherlands), it was possible to manipulate blood flow to obtain a desired pressure level. Data were saved through Sophisticate. A software driver (Phidget, Clgary, AB, Canada) was used as interface between the hardware and the software. 

The scan protocol was performed in two steps. The pump was regulated to provide an arterial pressure of 40–50 mmHg resulting in a flow of 70–140 mL/min for the first part and an arterial pressure of 80–100 mmHg resulting in a flow of 140–280 mL/min for the second part. Arterial and venous samples were analysed during perfusion to monitor blood oxygenation, pH, electrolyte, and lactate levels using the ABL800 Flex (Radiometer, Copenhagen, Denmark). The haemoglobin concentration was sub-physiological (approximately 6.6 mmol/L) due to dilution with saline during priming. pH and electrolyte values were for all experiments within physiological levels. pO_2_ values of the perfusate were in general high (around 65 kPa), except for one experiment where perfusate pO_2_ was around 7.5 kPa.

### 2.2. PET/MRI Examination

PET/MRI examination was performed using a clinical 3.0 T Signa PET/MRI scanner (GE Healthcare, Milwaukee, WI, USA). The organ chamber was positioned horizontally in an 8-channel head MRI-coil (GE Healthcare, Milwaukee, WI, USA). The renal anatomy was assessed using both axial and coronal T1-weighted 3D LAVA Flex imaging, as well as a with a T2-weighted coronal gradient-echo (GRE) sequence with fat suppression. The imaging parameters for coronal 3D LAVA Flex were: TR = 5.7 ms, two TEs per scan, TE1/TE2 = 1.1 ms/2.2 ms, flip angle = 12°, FOV = 40 × 40 cm^2^, matrix = 320 × 256, 1 slab, 32 locations per slab, slice thickness = 3 mm, NEX = 6. The imaging parameters for axial 3D LAVA Flex were the same as for the coronal acquisition, with the exception of the following parameters: TR = 5.6 ms, FOV = 40 × 32 cm^2^, locations per slab = 48. 3D LAVA Flex was used to reconstruct Water, Fat, In- and Out-Phase images. The imaging parameters for GRE with fat suppression were: TR = 2.5 s, TE = 102 ms, echo train length = 16, flip angle = 111°, FOV = 34 × 34 cm^2^, matrix = 448 × 256, 13 slices, slice thickness = 4 mm, slice spacing = 0.4 mm, NEX = 6.

Two sets of perfusion measurements were performed using H_2_^15^O PET; one measurement for each pump flow rate. 75 MBq H_2_^15^O was diluted in 20 mL saline and injected with a flow rate of 1 mL/s. The PET acquisitions each had a duration of 7 min and were reconstructed as a dynamic image series with 26 frames (1 × 10 s, 8 × 5 s, 4 × 10 s, 2 × 15 s, 3 × 20 s, and 8 × 30 s) in 2.8 × 2.8 × 2.8 mm^3^ isotropic voxels using the VuePointFX SharpIR algorithm with all common corrections and a 3 mm Gaussian postfilter applied. The pump settings were adjusted immediately after the termination of the first PET scan, and the second PET scan was performed 30 min afterwards. Simultaneously with each PET acquisition, coronal BOLD MRI was acquired utilizing a multi-echo (fast spoiled gradient echo) FSPGR sequence to assess renal tissue oxygenation. The imaging parameters for BOLD MRI were: TR = 300 ms, 16 echos with TE = 1.14–27.66 ms in increments of 1.77 ms, flip angle = 60°, FOV = 42 × 34 cm^2^, matrix = 192 × 192, 3 slices, slice thickness = 10 mm, slice spacing = 2 mm [13]. Prior to BOLD MRI acquisition, optimal shimming was verified by B0 mapping, ensuring a homogeneous field across the renal tissue necessary for accurate determination of the T2* relaxation time constant [14]. B0 mapping was acquired utilizing a 3D Iterative Decomposition of water and fat with Echo Asymmetry and Least squares estimation (IDEAL) IQ sequence covering the entirety of the ex vivo organ chamber.

### 2.3. Data Analysis

**Figure 2:**
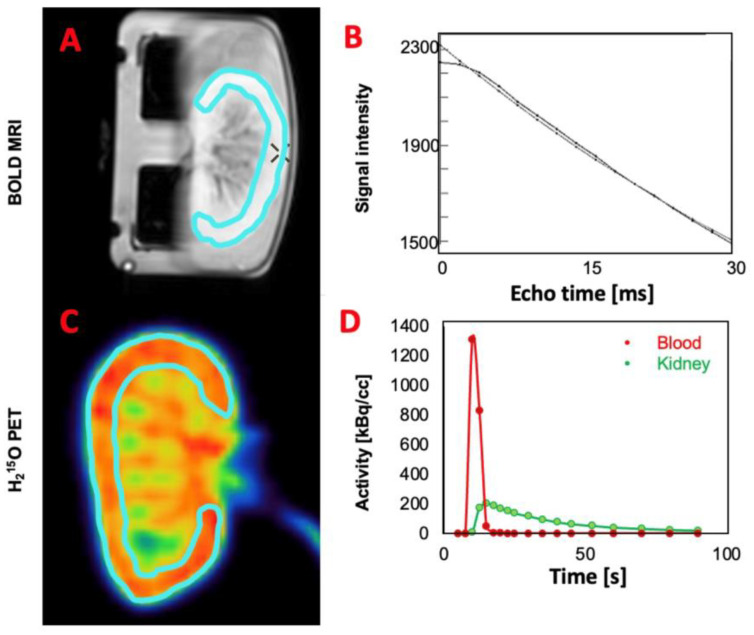
Examples of BOLD MRI and PET images. (**A**) Example of a blood-oxygenation-level-dependent (BOLD) MRI image and a manually drawn region of interest (ROI) defining the renal cortex. (**B**) Example of the mono-exponential fitting procedure to determine voxel-vise T2* (BOLD) values, as performed in the scanner interface. (**C**) Example of a H_2_^15^O uptake image acquired with PET and a manually drawn ROI defining the renal cortex. (**D**) Example of time–activity curves following injection of H_2_^15^O (blood refers to the activity in the inflow tube, which is not shown on the picture, and kidney refers to the activity in the ROI).

T2* mapping was performed using the BOLD MRI data directly on the scanner, by means of mono-exponential fitting of the signal intensity with respect to the echo time (Figure 2A,B) [14].

Additional BOLD MRI analysis, including ROI delineation of the cortical tissue, was performed in PMOD Version 4.0 (PMOD Technologies LLC, Zürich, Switzerland) [15]. H_2_^15^O scans were analysed using a standard one-tissue compartment model with two rate constants corresponding to uptake, k1, and clearance, k2. RBP was estimated as the clearance rate, k2, multiplied by the physiological partition coefficient, *p* = 0.94 mL/g [16]. The blood input function was measured in transaxial images by averaging three circular regions of interest (ROIs) drawn on the tube entering the organ chamber and corrected for partial volume effects using the known inner diameter of the tube. Cortical ROIs were drawn on summed coronal images in the centre slice only. PET-data were also analysed using PMOD. Statistical analysis included simple data plots and linear regression analysis performed in Excel (Microsoft^®^ Excel, version 16.75.2, USA).

## 3. Results

The complete protocol was successfully performed on seven kidneys. The mean RBP measured using H_2_^15^O PET was during the first flow setting 0.62 ± 0.13 mL/min/g (mean ± SD) and during the second flow setting 1.2 ± 0.30 mL/min/g. The mean RPO measured using BOLD MRI was during the first flow setting 37.3 ± 11.2 ms and during the second flow setting 41.5 ± 10.4 ms. Examples of a BOLD MRI image and the associated relationship between echo time and signal intensity for measurements of T2* are presented in Figure 2A,B. An example of a H_2_^15^O uptake image acquired with PET and associated time–activity curves following injection of H_2_^15^O are presented in Figure 2C,D. 

Kidney weights and relative changes in RABF resulting from increasing pump flow as well as the corresponding relative changes in RBP are presented in Table 1.

**Figure 3:**
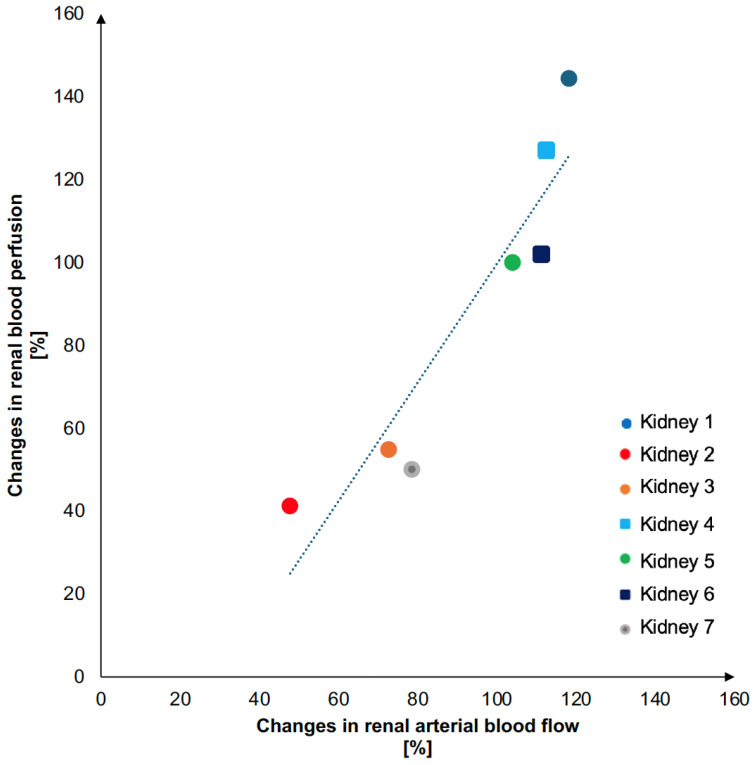
Graphical visualization of the association between changes in RABF and RBP. The graph visualizes the linear association between percentage changes in renal arterial blood flow (pump flow) caused by changing the pump settings, and renal blood perfusion (based on H_2_^15^O PET dynamic uptake), with two successive pump flow settings.

We found an agreement between the relative increase in RABP and the corresponding increase in RBP with the coefficient of determination (r2) of 0.87 (Figure 3). The slope of the fitted linear regression was 1.43.

**Figure 4:**
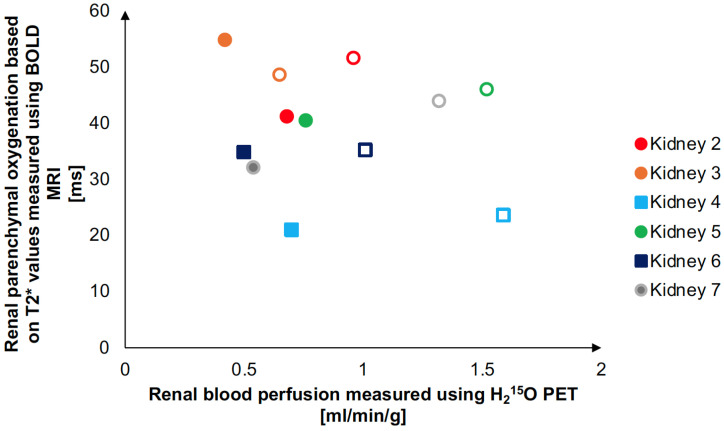
Graphical visualization of the association between RBP and RPO. The graph visualizes the association between the renal blood perfusion measured in renal cortex (based on H_2_^15^O PET dynamic uptake measurements) and the simultaneous measurement of renal parenchymal oxygenation (MRI measured apparent relaxation time in renal cortex, T2*, BOLD MRI). Filled symbols: flow setting 1, open symbols: flow setting 2.

Measured RPO, quantified based on the T2* values obtained by the analysis of the BOLD MRI images, were plotted against measured RBP (Figure 4).

## 4. Discussion

We introduced H_2_^15^O PET/MRI as an investigational method for simultaneous determination of RBP and RPO during ex vivo perfusion of isolated kidneys. We found a positive correlation between changes in delivered blood flow from the perfusion pump and changes in measured RBP using PET. Furthermore, we were able to measure RPO using BOLD MRI simultaneously with each PET image acquisition.

We have previously demonstrated the feasibility of renal anatomy, perfusion and metabolism examination using hyperpolarized [1-^13^C]pyruvate MRI during ex vivo kidney perfusion [17]. The addition of H_2_^15^O PET simultaneously with MRI enables the performance of a validated renal perfusion measurement without administration of gadolinium, which today is contraindicated in patients with reduced kidney function due to the strong association to nephrogenic systemic fibrosis (NSF). Although the use of group II gadolinium-based agents is associated with a low risk of NSF [18], many hospitals still avoid gadolinium-based contrast agents in patients with reduced kidney function reducing diagnostic and research opportunities. Therefore, introduction of H_2_^15^O PET for renal perfusion measurements simultaneously with MRI is highly relevant. 

The T2* values measured in our study are within the same level as previously measured using ex vivo kidney perfusion [10]. The lack of a correlation between RPO and RBP at different flow settings is most likely because the oxygen supply meets the need of the kidneys, even at the lower perfusion rate. Furthermore, it has previously been demonstrated that an abrupt total stop in perfusion does not lead to a corresponding decrease in T2* until 20 min without perfusion [10,19]. 

A positive correlation between changes in delivered blood flow and H_2_^15^O PET measured RBP was found. The slope of the fitted linear regression was 1.43, indicating a larger increase of RBP compared to delivered RABF. Since the delivered blood flow includes the total renal blood supply and the H_2_^15^O PET analysis is based on ROI’s defining renal cortex, blood shunting between cortex and medulla due to haemodynamic changes is a plausible explanation of the measured difference [14]. 

In general, the use of combined PET/MRI as imaging technology is increasing. Espes et al. found a positive correlation between pancreatic perfusion measured by MRI and H_2_^15^O PET [20], while Puig et al. demonstrated a positive correlation of H_2_^15^O PET and arterial spin labelling (ASL) MRI measurements of regional cerebral blood flow [21]. However, the use of PET/MRI in kidney research has been limited. Geist et al. performed fluorodeoxyglucose-PET/MRI in healthy subjects for assessment of various kidney functional parameters, including glomerular filtration rate, effective renal plasma flow, split function, mean transit time, and outflow efficiency [22,23]. These studies differ from our study with the MRI sequences only being used as guidance for PET analyses. To our knowledge, this study is the first study using H_2_^15^O PET/MRI in kidney research for combined measurements of RBP and RPO. 

MRI-based arterial spin labelling (ASL) is an alternative to PET-based perfusion and the relation between these modalities needs further investigations. For the present study we did not have access to the appropriate MR software sequence necessary for performing ASL in the kidneys. However, a positive correlation between ASL and H_2_^15^O PET measured RBP could be expected due to the similar flow values produced by the two methods in, e.g., the brain [24]. Corroborating this expectation is the absence of motion in ex vivo experiments, which would normally be of concern for in vivo renal ASL measurements. In addition to ASL MRI as a second perfusion measurement, it would be interesting to perform and compare both the ASL MRI and the H_2_^15^O PET perfusion measurements with Dynamic Contrast Enhancement (DCE) MRI to assess renal perfusion. However, despite comparable results, a higher reproducibility of ASL MRI for renal imaging has previously been demonstrated when comparing ASL MRI and DCE MRI [25,26]. Furthermore, ASL MRI might be preferable due to the need of contrast administrating for the performance of DCE MRI. Additionally, the performance of phase contrast (PC) MRI would be interesting. PC MRI measurements would be expected to correlate positively with the pump-delivered RABF and to have a similar correlation with the PET-measured RBP, as found between the pump-delivered RABF and PET-measured RBP in this study. 

Schutter et al. previously demonstrated the feasibility of performing MRI during warm ex vivo kidney perfusion and introduced the potential of pre-transplant evaluation of donor kidneys during NMP using non-invasive imaging [10]. By introducing H_2_^15^O PET/MRI during NMP, we added the opportunity to perform high quality perfusion measurements simultaneously with MRI, which could be highly relevant for both organ quality evaluation and in the future for evaluating changes in renal perfusion caused by drug administration during NMP. In addition to the potential of H_2_^15^O PET/MRI for pre-transplant organ monitoring, the introduction of H_2_^15^O PET/MRI in kidney research brings more opportunities as a tool for studying renal physiology in both healthy and diseased kidneys, which potentially could improve renal diagnostics. 

Some limitations should be addressed. The range of flow values used with the pump system were slightly below the physiological ranges of RBF, both in relation to healthy humans and pigs [27,28]. However, the high resistance in the long tubing system and possibly also the kidney resulted in high perfusion pressures when pump pressure was increased to higher levels. The lower flow would result in decreased signal-to-noise ratio However, this should not affect the reliability or accuracy of the perfusion measurements. As an example, H_2_^15^O PET has previously been used in perfusion-impaired kidneys [29].

Regarding MRI, magnetic field inhomogeneities occurred due to the susceptibility difference between air and tissue at the surface of the perfused kidney. This phenomenon is particularly important in BOLD MRI sequences because T2*-weighted sequences are inherently sensitive to spatial distortions in the magnetic field. Therefore, optimal shimming is important but can be hard to achieve. In the present study, the acquisition of magnetic field maps was utilized to exclude areas from the kidney ROI delineation, where field distortions were present, producing more accurate T2* measures. In future studies, an MRI-optimized perfusion chamber should be used to completely surround the kidney with liquid, in order to eliminate any air-to-tissue interfaces. This, possibly in conjunction with retrospective field inhomogeneity correction, should serve to give accurate T2* estimation throughout the entire renal cortical tissue.

The use of the twelve-layer concentric objects method (TLCO) for BOLD MRI analysis could probably have improved the accuracy of the BOLD MRI results [30]. However, since the resolution of the H_2_^15^O PET data does not support such detailed division, the analysis was based on manually drawn ROIs to secure the highest degree of comparability. The analysis and comparison between RBP in renal medulla were furthermore limited by the resolution obtainable by H_2_^15^O PET, and therefore not included in this study. 

Quantification of RBP with H_2_^15^O PET using a single-tissue compartment model is well-established and validated using microspheres [31]. The current study utilizes the tracer clearance rate, which is less affected by partial volume effects and tissue heterogeneity but instead is affected by the glomerular filtrate rate (GFR), which has been estimated to constitute approximately 10% of the clearance [16].

In this study, we demonstrated the feasibility of combined H_2_^15^O PET/MRI during NMP of isolated porcine kidneys. In the future, we initially aim to introduce combined H_2_^15^O PET/MRI in healthy humans, focusing on repeatability and reproducibility of both H_2_^15^O PET and MRI. Additionally, as mentioned previously in the discussion section, we aim to incorporate more MRI sequences, revealing measurements of more renal physiological parameters. Based on further studies, we believe that combined H_2_^15^O PET/MRI might contribute with relevant clinical information, thus potentially enhancing renal diagnostics in the future. 

## 5. Conclusions

We demonstrated the feasibility of combined H_2_^15^O PET/MRI during NMP of isolated porcine kidneys and found a positive correlation between changes in delivered blood flow and changes in measured RBP using H_2_^15^O PET. Furthermore, we were able to measure RPO using BOLD MRI simultaneously with each PET image acquisition.

## Figures and Tables

**Table 1:** Kidney weight and changes in pump flow and renal blood perfusion during the experiment. Kidney weights and percentage increases in delivered blood flow (pump flow) and the corresponding changes in renal perfusion (based on H215O dynamic uptake), with two successive pump flow settings.

Kidney (Number)	Weight (g)	Pump Flow Increase (%)	Renal Blood Perfusion Increase (%)
1	107	79	50
2	165	48	41
3	170	73	54
4	169	113	127
5	190	104	100
6	242	111	102
7	213	118	144

## Data Availability

Data supporting the findings of the study are available upon request from the corresponding author.

## References

[1] Webster A.C., Nagler E.V., Morton R.L., Masson P. (2017). Chronic Kidney Disease. Lancet.

[2] Vaidya S.R., Aeddula N.R. (2022). Chronic Renal Failure.

[3] Khatir D.S., Pedersen M., Jespersen B., Buus N.H. (2013). Reproducibility of MRI renal artery blood flow and BOLD measurements in patients with chronic kidney disease and healthy controls. J. Magn. Reson. Imaging.

[4] Green M.A., Hutchins G.D. (2011). Positron Emission Tomography (PET) Assessment of Renal Perfusion. Semin. Nephrol..

[5] Päivärinta J., Koivuviita N., Oikonen V., Iida H., Liukko K., Manner I., Löyttyniemi E., Nuutila P., Metsärinne K. (2018). The renal blood flow reserve in healthy humans and patients with atherosclerotic renovascular disease measured by positron emission tomography using [15O]H_2_O. EJNMMI Res..

[6] Eckerbom P., Hansell P., Cox E.F., Buchanan C., Weis J., Palm F., Francis S.T., Liss P. (2019). Multiparametric assessment of renal physiology in healthy volunteers using noninvasive magnetic resonance imaging. Am. J. Physiol. Physiol..

[7] Ebrahimi B., Textor S.C., Lerman L.O. (2014). Renal Relevant Radiology. Clin. J. Am. Soc. Nephrol..

[8] de Bazelaire C., Alsop D.C., George D., Pedrosa I., Wang Y., Michaelson M.D., Rofsky N.M. (2008). Magnetic Resonance Imaging–Measured Blood Flow Change after Antiangiogenic Therapy with PTK787/ZK 222584 Correlates with Clinical Outcome in Metastatic Renal Cell Carcinoma. Clin. Cancer Res..

[9] Gloviczki M.L., Glockner J.F., Lerman L.O., McKusick M.A., Misra S., Grande J.P., Textor S.C. (2010). Preserved Oxygenation Despite Reduced Blood Flow in Poststenotic Kidneys in Human Atherosclerotic Renal Artery Stenosis. Hypertension.

[10] Schutter R., van Varsseveld O.C., Lantinga V.A., Pool M.B.F., Hamelink T.H., Potze J.H., Leuvenink H.G.D., Laustsen C., Borra R.J.H., Moers C. (2022). Magnetic resonance imaging during warm ex vivo kidney perfusion. Artif. Organs.

[11] Frisch K., Mortensen F.V., Munk O.L., Gormsen L.C., Alstrup A.K.O. (2022). N-(4-[18F]fluorobenzyl)cholylglycine, a potential tracer for positron emission tomography of enterohepatic circulation and drug-induced inhibition of ileal bile acid transport. A proof-of-concept PET/CT study in pigs. Nucl. Med. Biol..

[12] du Sert N.P., Hurst V., Ahluwalia A., Alam S., Avey M.T., Baker M., Browne W.J., Clark A., Cuthill I.C., Dirnagl U. (2020). The ARRIVE guidelines 2.0: Updated guidelines for reporting animal research. Exp. Physiol..

[13] Li L.-P., Halter S., Prasad P.V. (2008). Blood Oxygen Level-Dependent MR Imaging of the Kidneys. Magn. Reson. Imaging Clin. N. Am..

[14] Bane O., Mendichovszky I.A., Milani B., Dekkers I.A., Deux J.-F., Eckerbom P., Grenier N., Hall M.E., Inoue T., Laustsen C. (2019). Consensus-based technical recommendations for clinical translation of renal BOLD MRI. Magn. Reson. Mater. Phys. Biol. Med..

[15] Niendorf T., Pohlmann A., Arakelyan K., Flemming B., Cantow K., Hentschel J., Grosenick D., Ladwig M., Reimann H., Klix S. (2014). How bold is blood oxygenation level-dependent (BOLD) magnetic resonance imaging of the kidney? Opportunities, challenges and future directions. Acta Physiol..

[16] Kudomi N., Koivuviita N., Liukko K.E., Oikonen V.J., Tolvanen T., Iida H., Tertti R., Metsärinne K., Iozzo P., Nuutila P. (2008). Parametric renal blood flow imaging using [15O]H_2_O and PET. Eur. J. Nucl. Med..

[17] Mariager C., Hansen E.S.S., Bech S.K., Munk A., Kjærgaard U., Lyhne M.D., Søberg K., Nielsen P.F., Ringgaard S., Laustsen C. (2020). Graft assessment of the ex vivo perfused porcine kidney using hyperpolarized [1-^13^C]pyruvate. Magn. Reson. Med..

[18] Woolen S.A., Shankar P.R., Gagnier J.J., MacEachern M.P., Singer L., Davenport M.S. (2020). Risk of Nephrogenic Systemic Fibrosis in Patients With Stage 4 or 5 Chronic Kidney Disease Receiving a Group II Gadolinium-Based Contrast Agent. JAMA Intern. Med..

[19] Evans R.G., Ince C., Joles J.A., Smith D.W., May C.N., O’Connor P.M., Gardiner B.S. (2013). Haemodynamic influences on kidney oxygenation: Clinical implications of integrative physiology. Clin. Exp. Pharmacol. Physiol..

[20] Espes D., Manell E., Rydén A., Carlbom L., Weis J., Jensen-Waern M., Jansson L., Eriksson O. (2019). Pancreatic perfusion and its response to glucose as measured by simultaneous PET/MRI. Acta Diabetol..

[21] Puig O., Henriksen O.M., Vestergaard M.B., E Hansen A., Andersen F.L., Ladefoged C.N., Rostrup E., Larsson H.B., Lindberg U., Law I. (2019). Comparison of simultaneous arterial spin labeling MRI and ^15^O-H_2_O PET measurements of regional cerebral blood flow in rest and altered perfusion states. J. Cereb. Blood Flow Metab..

[22] Geist B.K., Baltzer P., Fueger B., Hamboeck M., Nakuz T., Papp L., Rasul S., Sundar L.K.S., Hacker M., Staudenherz A. (2018). Assessing the kidney function parameters glomerular filtration rate and effective renal plasma flow with dynamic FDG-PET/MRI in healthy subjects. EJNMMI Res..

[23] Geist B.K., Baltzer P., Fueger B., Hamboeck M., Nakuz T., Papp L., Rasul S., Sundar L.K.S., Hacker M., Staudenherz A. (2019). Assessment of the kidney function parameters split function, mean transit time, and outflow efficiency using dynamic FDG-PET/MRI in healthy subjects. Eur. J. Hybrid Imaging.

[24] Zhang K., Herzog H., Mauler J., Filss C., Okell T.W., Kops E.R., Tellmann L., Fischer T., Brocke B., Sturm W. (2014). Comparison of Cerebral Blood Flow Acquired by Simultaneous [^15^O]Water Positron Emission Tomography and Arterial Spin Labeling Magnetic Resonance Imaging. J. Cereb. Blood Flow Metab..

[25] Cutajar M., Thomas D.L., Hales P.W., Banks T., Clark C.A., Gordon I. (2014). Comparison of ASL and DCE MRI for the non-invasive measurement of renal blood flow: Quantification and reproducibility. Eur. Radiol..

[26] Winter J.D., Lawrence K.S.S., Cheng H.M. (2011). Quantification of renal perfusion: Comparison of arterial spin labeling and dynamic contrast-enhanced MRI. J. Magn. Reson. Imaging.

[27] Dalal R., Bruss Z.S., Sehdev J.S. (2022). Physiology, Renal Blood Flow and Filtration.

[28] Hannon J.P., Bossone C.A., Wade C.E. (1990). Normal physiological values for conscious pigs used in biomedical research. Lab. Anim. Sci..

[29] Alpert N.M., Rabito C.A., Correia D.J.A., Babich J.W., Littman B.H., Tompkins R.G., Rubin N.T., Rubin R.H., Fischman A.J. (2002). Mapping of local renal blood flow with PET and H(2)(15)O. J. Nucl. Med..

[30] Milani B., Ansaloni A., Sousa-Guimaraes S., Vakilzadeh N., Piskunowicz M., Vogt B., Stuber M., Burnier M., Pruijm M. (2016). Reduction of cortical oxygenation in chronic kidney disease: Evidence obtained with a new analysis method of blood oxygenation level-dependent magnetic resonance imaging. Nephrol. Dial. Transplant..

[31] Juillard L., Janier M.F., Fouque D., Lionnet M., Le Bars D., Cinotti L., Barthez P., Gharib C., Laville M. (2000). Renal blood flow measurement by positron emission tomography using 15O-labeled water. Kidney Int..

